# CSF peptidomics reveals an IGF2-derived peptide as a candidate biomarker for neurosyphilis diagnosis

**DOI:** 10.3389/fmicb.2026.1931675

**Published:** 2026-09-04

**Authors:** Yue Yin, Xinru Li, Jiayin Han, Jiatong Li, Lijiao Zeng, Jianfeng Lan, Weizhen Weng, Yanhua Liang, Ying Zhang, Rongrong Zou, Shuai Wu, Guoyu Tan, Shenghong Tang, Jing Yuan

**Affiliations:** 1 Shenzhen Key Laboratory of Pathogen and Immunity, State Key Discipline of Infectious Disease, Shenzhen Third People's Hospital, Second Hospital Affiliated with Southern University of Science and Technology, Shenzhen, China; 2 School of Public Health, Shenzhen University Medical School, Shenzhen University, Shenzhen, China; 3 Department of Infectious Diseases, The Affiliated Hospital of Southwest Medical University, Luzhou, China; 4 Shantou University Medical College, Shantou University, Shantou, China

**Keywords:** cerebrospinal fluid, diagnosis, neurosyphilis, peptidomics, *Treponema pallidum*

## Abstract

**Background:**

Neurosyphilis (NS) diagnosis is challenging due to the cerebrospinal fluid-Venereal Disease Research Laboratory (CSF-VDRL) assay has limited sensitivity, whereas CSF abnormalities lack sufficient specificity.

**Methods:**

To identify novel CSF peptide biomarkers for NS, we performed comparative peptidomic analysis of CSF samples from 51 individuals across four groups: NS, syphilis/non-NS (NNS), infectious brain diseases without syphilis (IBD), and non-infectious brain disorders without syphilis (NIBD). Peptides were profiled by high-resolution liquid chromatography-tandem mass spectrometry (LC-MS/MS). Bioinformatic analysis and machine learning identified candidate biomarkers, validated by parallel reaction monitoring (PRM) in an independent cohort (*n* = 23).

**Results:**

A total of 4,738 peptides were identified. The insulin-like growth factor 2 (IGF2)-derived peptide GHVLAKELE showed strong discriminative performance: area under the curve (AUC) values of 0.914 (NS vs. NNS), 0.964 (NS vs. IBD), and 0.878 (NS vs. NIBD).

**Conclusion:**

This first systematic CSF peptidomic characterization of neurosyphilis identifies GHVLAKELE as a promising diagnostic biomarker.

## Introduction

*Treponema pallidum (T. pallidum)* can invade the central nervous system (CNS) at early stages of infection, leading to NS, a condition with highly heterogeneous clinical manifestations ranging from asymptomatic disease to severe neurological impairment (Radolf et al., 2016). Despite advances in clinical diagnostics, the accurate identification of NS remains challenging.

Current diagnostic strategies rely primarily on CSF analysis, including pleocytosis, elevated protein levels, and reactivity in the CSF-VDRL test. Although the CSF-VDRL assay is highly specific, its limited sensitivity results in a substantial proportion of false-negative cases. Conversely, other CSF abnormalities lack specificity, as similar changes can be observed in a variety of infectious and non-infectious neurological disorders (Golden et al., 2003; Ropper, 2019). This diagnostic uncertainty may lead to delayed treatment or unnecessary intervention.

Peptidomics has emerged as a powerful approach for profiling endogenous peptides and has shown promise in the identification of disease-associated biomarkers across multiple conditions (Antwi et al., 2009; Liu et al., 2016; Neves et al., 2021; Huan et al., 2022; Palanski et al., 2022). Compared with conventional proteomics, peptidomics focuses on low-molecular-weight peptides that may reflect proteolytic activity and dynamic pathological processes. The biological importance of peptide-level regulation is increasingly recognized. For instance, a recent study demonstrated that a hepatic micropeptide modulates mitochondrial RNA processing machinery in hepatocellular carcinoma, highlighting how small peptides can influence fundamental cellular pathways (Zhu et al., 2025). Similarly, another study revealed that HIF regulates multiple translated endogenous retroviruses, with direct implications for cancer immunotherapy, further underscoring the functional and clinical relevance of non-canonical peptides (Jiang et al., 2025). In the context of fibrotic diseases, immunopeptidome profiling in pulmonary fibrosis has been shown to provide a platform for identifying therapeutic targets, demonstrating the translational potential of peptide-centric approaches beyond oncology (Bai et al., 2026). These examples illustrate the broad potential of peptidomics beyond oncology, extending to diseases where proteolytic activity and peptide signaling are critical. This feature is particularly relevant for neurological diseases, where protease activity, neuroinflammation, and blood-brain barrier disruption contribute to disease progression. Given that NS involves both infection and neuroinflammation, peptidomics may offer unique insights into its molecular features. However, whether NS is associated with a distinct CSF peptide profile remains unknown.

To test this hypothesis, we performed a comparative peptidomic analysis of CSF samples from four clinically distinct groups, including NS, NNS, IBD and NIBD. This design allowed us to distinguish peptide alterations associated with CNS invasion from those related to systemic infection or non-specific neurological conditions. Candidate biomarkers were further evaluated using machine learning and validated in an independent cohort using targeted mass spectrometry.

## Materials and methods

### Ethics approval and patient consent statement

The protocol for this study was reviewed and approved by the Institutional Review Board (IRB) of Shenzhen Third People's Hospital (Approval ID: 2026-052-01). Prior to any sample collection, written informed consent was obtained from all participants following the hospital's IRB-approved procedures for lumbar puncture and CSF collection.

### Study design and case definition

We enrolled the following patient cohorts: NS, NNS, IBD, and NIBD. NS patients were defined as first-admission, treatment-naïve individuals diagnosed with early neurosyphilis, according to the Chinese National Diagnostic Criteria for Syphilis (WS 273-2018). The diagnosis was based on comprehensive evaluation of clinical manifestations, CSF test [positive CSF-*Treponema pallidum* particle agglutination assay (TPPA)/Toluidine Red Unheated Serum Test (TRUST), CSF White Blood Cell (WBC) count (>5 cells/μL), CSF protein levels (>45 mg/dL)], positive serum TRUST, and assessment by experienced infectious disease specialists. Notably, the CSF-VDRL test was not performed in this study; instead, CSF-TRUST was used as the routine serological assay for CSF examination, consistent with standard practice in many Chinese tertiary care centers. NNS was defined as negative CSF-TRUST results in syphilis patients who lacked both CSF WBC count (>5 cells/μL) and elevated CSF protein levels (>45 mg/dL), and who had no characteristic symptoms or signs of NS. The IBD and NIBD cohorts comprised patients who presented with neurological symptoms and were ultimately diagnosed with other CNS disorders, including both infectious and noninfectious brain diseases and unrelated to syphilis, The IBD cohort predominantly comprised patients with viral meningoencephalitis (e.g., herpes simplex virus, varicella-zoster virus), while the NIBD cohort included patients with hepatic encephalopathy, Guillain Barre Syndrome, and anxiety disorders. Exclusion criteria included (1) traumatic lumbar puncture; (2) prior anti-syphilitic treatment within the past 3 months; (3) pregnancy or lactation; (4) concurrent human immunodeficiency virus (HIV) infection or other immunocompromising conditions; (5) age < 18 years; and (6) inability to provide informed consent. Demographic and clinical data—age, sex, and diagnosis—were recorded. Full patient metadata are available in Supplementary Table S1.

### Cerebrospinal fluid sample collection

According to international guidelines, CSF samples were obtained by lumbar puncture (L3/L4, L4/L5, or L5/S1). Each sample was collected directly into a sterile polypropylene tube. Samples with overt blood contamination (red blood cell count > 500/μL) were excluded. For all included samples, CSF was promptly aliquoted into 0.5 mL volumes and stored at −80 °C within 1 h to preserve sample integrity. To minimize proteolytic degradation, no exogenous protease inhibitors were added; instead, rapid processing and immediate low-temperature storage were employed as the primary preservation strategy. All samples underwent only one freeze-thaw cycle prior to peptide enrichment. Prior to peptidomic analysis, samples were inspected visually, and only clear, colorless samples were included in the study.

### Peptidomic sample preparation

Aliquot 5 mg of mesoporous particle material suspension (in 50% acetonitrile, v/v) into a 1.5 mL EP tube. Remove the sample from −80 °C, thaw on ice for 10 min, centrifuge at 4 °C, 18,000 g for 5 min, and transfer 1,200 μL of the supernatant to the mesoporous particle material suspension. After vortexing for 30 s, incubate the mixture on a horizontal shaker at room temperature (25 °C) at 1,500 rpm for 1 h. After incubation, centrifuge the sample at 10,000 g for 5 min at 4 °C, discard the supernatant, add 1 mL of ultrapure water, and wash on the horizontal shaker at 1,500 rpm for 5 min at room temperature (25 °C). Centrifuge at 10,000 g for 5 min at 4 °C, discard the supernatant, and repeat the wash three times. Finally, add 0.5 mL of 50% acetonitrile to the material, shake horizontally for 10 min, centrifuge at 10,000 g for 5 min, and collect the supernatant into a clean EP tube. This supernatant is the enriched peptide, which can be vacuum-dried for storage.

Dissolve the dried peptides in 100 μL of 0.1% trifluoroacetic acid (TFA), centrifuge at 12,000 g at room temperature for 10 min, and transfer the supernatant to a new EP tube. Activation: Add 50 μL of activation solution (100% methanol) to the StageTip and centrifuge at 1500 g for 1 min. Equilibration: Add 50 μL of desalting solution (0.1% TFA) to the StageTip and centrifuge at 1,500 g for 1 min, repeat once. Sample loading: Discard the waste in the EP tube, add 50 μL of peptide to the StageTip, and centrifuge at 1,500 g for 1 min. Desalting: Add 50 μL of desalting solution (0.1% TFA) to the StageTip and centrifuge at 1,500 g for 1 min, repeat once. Elution: Place the StageTip into a new EP tube, add 20 μL of elution solution (80% acetonitrile/0.1% TFA) to the StageTip, centrifuge at 750 g for 1 min, repeat once, combine the two elution fractions, and freeze-dry for future use.

### Mass spectrometry and data processing

The enriched endogenous peptides were dissolved in solvent A, and directly loaded onto a self-packed reversed-phase analytical column (25 cm length, 100 μm inner diameter). The mobile phases consisted of solvent A (0.1% formic acid and 2% acetonitrile in water) and solvent B (0.1% formic acid in acetonitrile). Peptide separation was performed on a NanoElute Ultra-High Performance Liquid Chromatography (UHPLC) system (Bruker Daltonics) using the following gradient program: 0–14 min, 6–24% solvent B; 14–16 min, 24–35% solvent B; 16–18 min, 35–80% solvent B; and 18–20 min, 80% solvent B, at a constant flow rate of 500 nL/min.

The eluted peptides were analyzed using a timsTOF Pro2 mass spectrometer equipped with a nano-electrospray ionization source. An electrospray voltage of 1.75 kV was applied. Both precursor and fragment ions were detected by the TOF analyzer. The timsTOF Pro2 was operated in data-independent parallel accumulation-serial fragmentation (dia-PASEF) mode. Full MS scans were acquired over an m/z range of 300–1,500, followed by 20 PASEF MS/MS scans per cycle. The MS/MS acquisition range was set to m/z 400–850 with an isolation window of 7 m/z.

Data-independent acquisition (DIA) raw data were processed using Spectronaut software (v18). Tandem mass spectra were searched against the Homo sapiens reference database (Homo_sapiens_9606_SP_20241202_openprot_20250627_seqkit_20250715.fasta; 686,192 entries) concatenated with a reverse decoy database. No enzyme cleavage specificity was specified, and the database search was performed using an unspecific/no-enzyme search strategy. Protein N-terminal acetylation and methionine oxidation were specified as variable modifications. The false discovery rate (FDR) was controlled at < 1% at both the peptide and peptide-spectrum match (PSM) levels. Protein-level FDR was controlled at < 100%.

### Random forest-based machine learning model

A random forest (RF) classifier was established to differentiate NS from NNS, leveraging peptidomic profiles obtained from the discovery cohort. The analytical workflow consisted of three sequential phases: (i) sample Statistics and Feature Selection, (ii) dataset Splitting, (iii) machine Learning Model Construction and Evaluation.

Dimensionality reduction and visualization of the data were performed using Uniform Manifold Approximation and Projection (UMAP). Features with a high missing rate (missing value proportion >50.0%) were filtered out, and the remaining missing values were imputed using the K-Nearest Neighbors (KNN) method. Features with zero variance were removed. Subsequently, the Pearson correlation coefficient between features was calculated, and for pairs of highly correlated features (*r* > 0.8), only the feature with the highest correlation to sample classification was retained. This process yielded 127 features for subsequent machine learning analysis. The dataset was then split using a stratified sampling method, the discovery cohort was partitioned into a training subset comprising 60% of the samples (NS, *n* = 8; NNS, *n* = 7) and an internal testing subset containing the remaining 40% (NS, *n* = 6; NNS, *n* = 5).

For the filtered data features, univariate feature analysis was employed to evaluate the discriminative ability of each feature with respect to sample classes. Features were ranked in ascending order according to their *p*-values derived from variance testing. Subsequently, incremental feature selection (IFS) was applied to identify the optimal feature subset. Model performance was assessed using the AUC and confusion matrices. Heatmaps were generated to visualize the variation of the selected features across different samples, and boxplots were used to illustrate the distribution differences of these peptides between sample classes.

### PRM and data processing

The enriched endogenous peptides were dissolved in solvent A, and directly loaded onto a home-made reversed-phase analytical column (15-cm length, 100 μm i.d.). The mobile phase consisted of solvent A (0.1% formic acid in water) and solvent B (0.1% formic acid, 80% acetonitrile/in water). Peptides were separated with the following gradient: 0–1.6 min, 4%−22.5%B; 1.6–2.0 min, 22.5%−35%B; 2.0–2.1 min, 35.0%−35.1%B; 2.1–2.3 min, 35.1%B; 2.3–9.2 min, 35.1%−35.2%B; 9.2–9.6 min, 35.2%−55.0%B; 9.6–10.1 min, 55.0%−99.0%B; 10.1–12.0 min, 99%B, and all at a constant flow rate of 200 nL/min on a Vanquish Neo UPLC system (ThermoFisher Scientific).

The separated peptides were analyzed in Orbitrap Astral with a nano-electrospray ion source. The electrospray voltage applied was 1900 V. Precursors were analyzed at the Orbitrap detector, and the fragments were analyzed at the Astral detector. The full MS scan resolution was set to 2,40,000 for a scan range of 400–1,250 m/z. The MS/MS scan at a resolution of 80,000. Automatic gain control (AGC) target was set at 100% for MS/MS, with a maximum injection time of 10 ms. Isolation window was set as 0.6 m/z.

The resulting MS data were processed using Skyline (v.22.2). Peptide settings: No enzyme was specified. The peptide length was set as 7–40. Transition settings: precursor charges were set as 2–6, ion charges were set as 1,2, ion types were set as b, y. The product ions were set as from ion 2 to the last ion −1, the ion match tolerance was set as 0.02 Da.

### Statistical analysis

All statistical analyses and data visualizations were performed using R software (version 4.0.3). Peptide abundance values were log2-transformed and mean-centered prior to downstream analyses. Pairwise differential expression analyses between two of the four clinical groups (NS, NNS, IBD, and NIBD) were performed using the R package limma (version 3.46.0) to identify peptides exhibiting significant abundance changes. Principal coordinate analysis (PCoA) based on peptides with valid quantitative values across samples was performed using the vegan package (version 2.5-7). Volcano plots and heatmaps were generated for data visualization and comparative analysis.

For the independent validation cohort, peptide intensities obtained by PRM-MS analysis were presented as mean ± standard error of the mean (SEM). Between-group comparisons were performed using the wilcox.test function (for non-parametric comparisons) or *t* test function (for parametric comparisons) in R, as appropriate. Receiver operating characteristic (ROC) curves were generated using the pROC package (version 1.17.0.1) to evaluate the discriminative performance of candidate peptides. Statistical significance thresholds were defined as follows: *P* ≤ 0.05 (^*^), *P* ≤ 0.01 (^**^), and *P* ≤ 0.001 (^***^).

Precursor proteins corresponding to the identified peptides were annotated by searching against the human reference proteome database (UniProtKB/Swiss-Prot) using Spectronaut software. Functional enrichment analyses of precursor proteins associated with differentially expressed peptides were performed using the online Database for Annotation, Visualization and Integrated Discovery (DAVID). Eggnog-mapper (v2.1.6) was used for Gene Ontology (GO) categories, including biological process, cellular component, and molecular function. Diamond (v2.0.11.149) was additionally used for Kyoto Encyclopedia of Genes and Genomes (KEGG) pathway enrichment analysis to evaluate the biological pathways associated with these precursor proteins.

## Results

### Peptidomic features of CSF from patients

**Figure 1:**
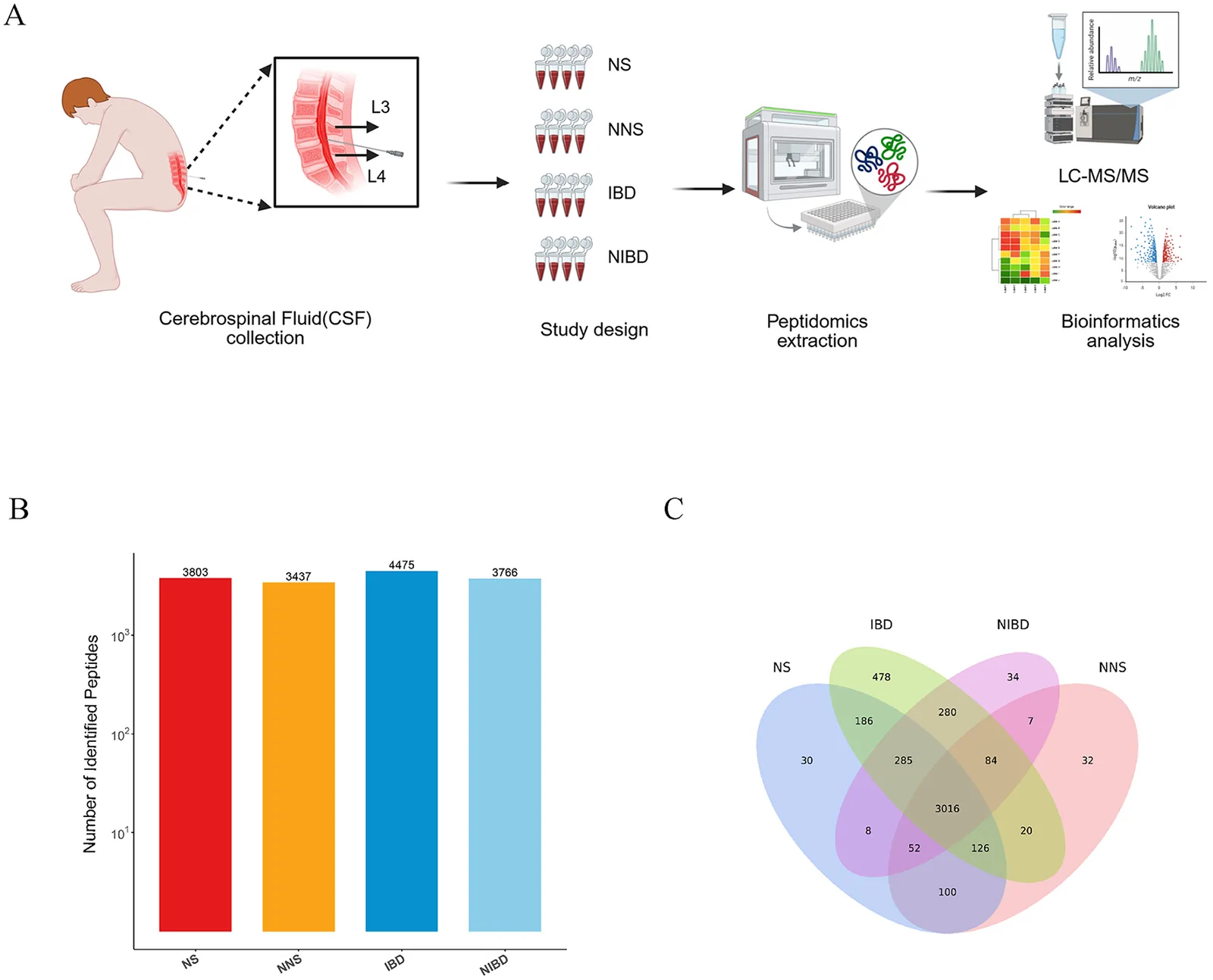
Study design and global CSF peptidome landscape. **(A)** Schematic illustration of the experimental workflow for quantitative peptidomic and subsequent bioinformatic analysis of CSF samples. Samples were collected from NS (*n* = 14), NNS (*n* = 12), IBD (*n* = 12), NIBD (*n* = 13). The isolated peptides from CSF samples were analyzed by LC-MS/MS using the data-independent acquisition (DIA) mode for identification and quantification. **(B)** Distribution of peptide numbers identified in CSF from NNS, NS, IBD, and NIBD samples. **(C)** Venn diagram of peptide identified in CSF from NNS, NS, IBD, and NIBD samples.

We collected 51 CSF samples from 14 NS, 12 NNS, 12 IBD and 13 NIBD patients for peptidomic identification (Figure 1A, Supplementary Table S1). We characterized the global peptidomic landscape of CSF across the four clinical groups. A total of 4,738 peptides were identified, with substantial overlap but also distinct group-specific patterns. The number of identified peptides varied across groups, indicating differences in CSF peptide composition among the clinical groups (Figures 1B, C). These findings indicate that CSF contains a complex and dynamic peptide repertoire that may reflect underlying disease processes.

### Peptidomic features of CSF from NS patients

**Figure 2:**
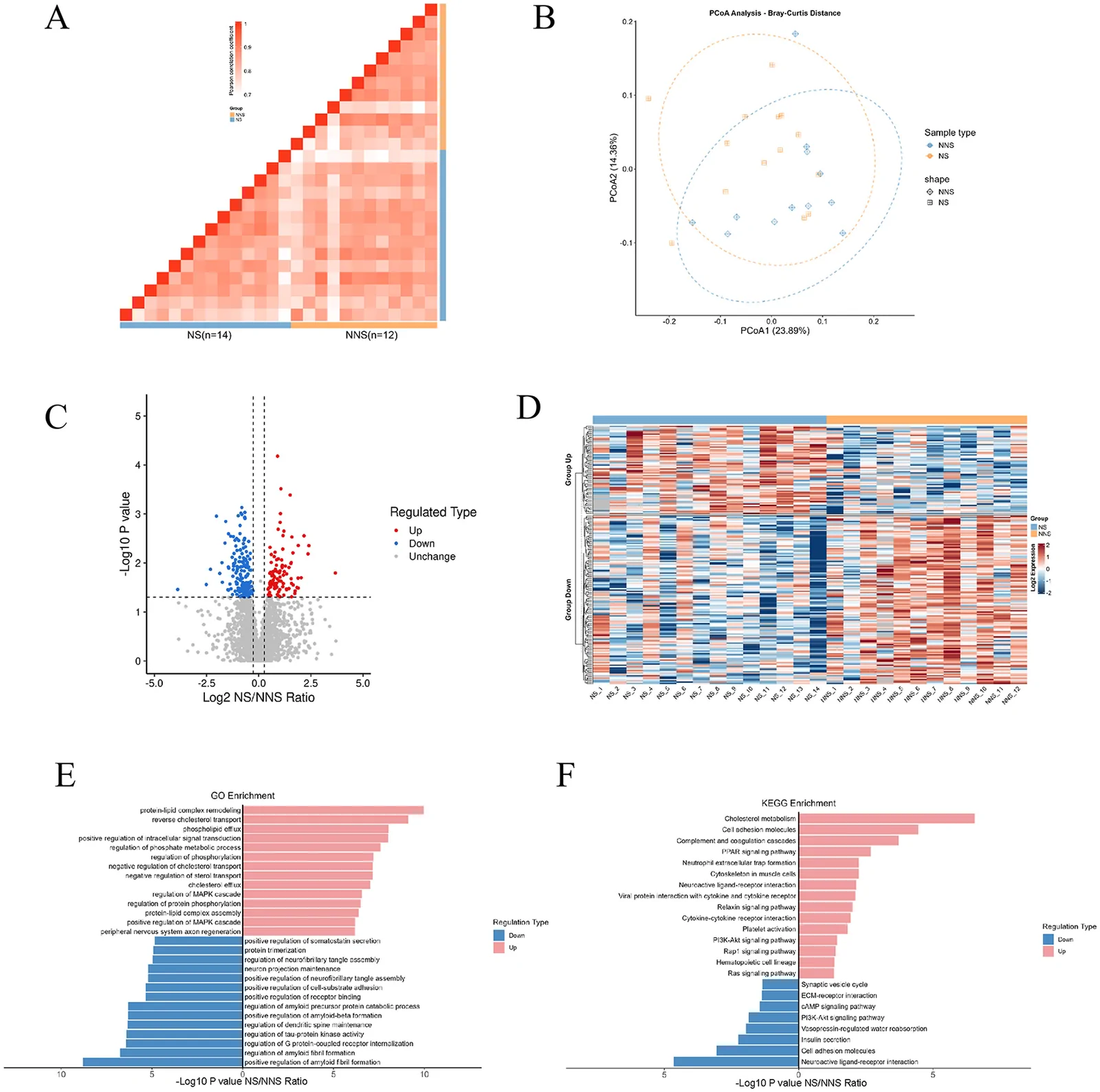
Differential peptidomic signatures and associated biological processes in NS. **(A)** Heatmap of overall relatedness of NNS and NS groups. Pearson correlation coefficients were calculated from relative peptide abundance patterns across all samples (range 0.7–1.00). **(B)** PCoA analysis of the peptidomic profile of NS (*n* = 14) and NNS samples (*n* = 12). **(C)** Differential expression analysis was conducted via limma, with results visualized as a volcano plot plotting −log10 (*p*-value) against log_2_(NS/NNS) fold change. Significantly differentially expressed peptides (FC cutoff: > 1.2 or < 1/1.2, *P* < 0.05) are highlighted in red (upregulated in NS) or blue (downregulated in NS), with unchanged peptides depicted in gray. Dashed lines mark the significance cutoffs. **(D)** Heatmap showing differentially expressed peptides in the CSF between NS (*n* = 14) and NNS (*n* = 12) samples. **(E)** Go enrichment analysis of differentially expressed peptides in CSF from NS and NNS patients, ranked by -log10 (*p*-value). Color indicates functional category. Expression changes of peptides that were significantly upregulated (shown in red text) or downregulated (shown in blue text) in NS compared to NNS samples are displayed. **(F)** KEGG enrichment analysis of differentially expressed peptides between NS and NNS samples was conducted using enrichment values [−log10 (*p*-value)]. Red and blue columns indicate upregulated and downregulated peptides in the NS vs. NNS comparison, respectively.

Overall CSF peptidomic abundance between the NS group and the NNS group exhibited strong positive correlations (Figure 2A). PCoA showed some overlap between the two groups (Figure 2B). Hierarchical clustering analysis (HCA) and volcano plot analysis revealed 255 differentially expressed peptides between the NS and NNS groups (Figures 2C, D, Supplementary Table S2). Comparative analysis between NS and NNS groups identified 255 differentially expressed peptides. Among these, 96 peptides were upregulated and 159 were downregulated in NS. Functional enrichment analysis of precursor proteins revealed that upregulated peptides were associated with biological processes related to axonal regeneration, intracellular signaling pathways [including mitogen-activated protein kinase (MAPK) activation], and lipid metabolism. These processes may reflect adaptive or compensatory responses to CNS injury. In contrast, downregulated peptides were enriched in pathways linked to neurodegenerative processes, including amyloid-beta formation and tau-related pathology. These associations suggest that neurosyphilis may share molecular features with neurodegenerative conditions, although the underlying mechanisms remain to be further investigated (Figure 2E). KEGG pathway analysis further indicated alterations in neuroendocrine signaling and synaptic function (Figure 2F). Together, these findings suggest that multiple interconnected biological processes may contribute to the neurological manifestations observed in NS.

### Potential biomarkers distinguishing NS from NNS patients

**Figure 3:**
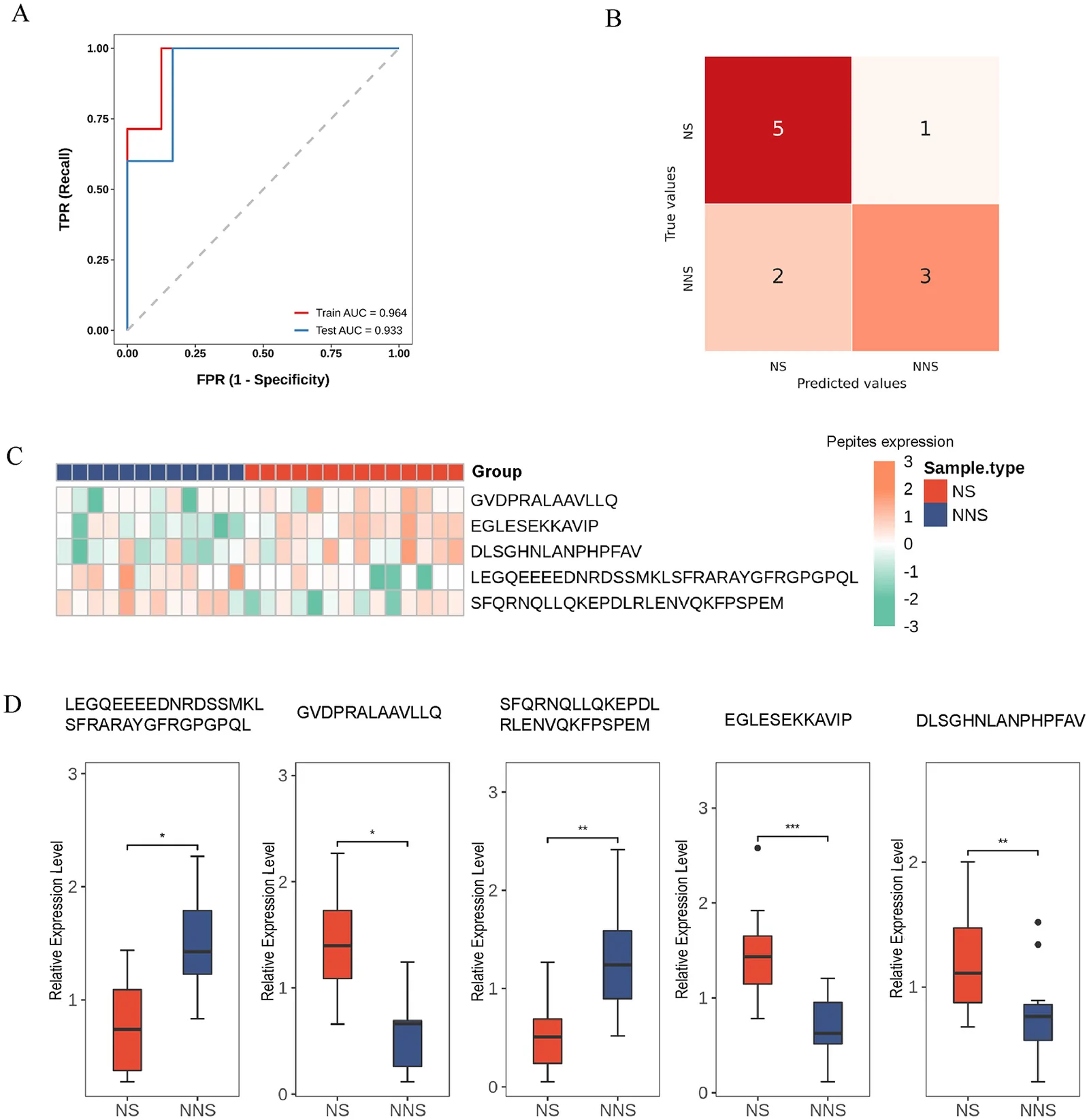
Machine learning identifies a diagnostic peptide signature of neurosyphilis from non-neurosyphilitic patients. **(A)** Receiver operating characteristic curve based on the RF model for classifying patients with neurosyphilis. The red line represents the values for the training group, and the blue line represents the values for the test group. **(B)** Confusion matrix showing the performance of the model for classifying patients with neurosyphilis on the test set. **(C)** Heatmap showing the variations in the selected model features across NS and NNS samples. **(D)** Boxplot showing the distribution differences of these peptides between NS and NNS groups. *P* ≤ 0.05 (*), *P* ≤ 0.01 (**), and *P* ≤ 0.001 (***).

In general, differentially expressed peptides between the NS and NNS groups may serve as unique markers. To determine the importance of these characteristic peptides in identifying NS, we constructed a random forest (RF) machine learning model based on differential peptide data from 14 NS and 12 NNS samples. An AUC of 0.964/0.933 was observed in the training/test cohort (Figure 3A). The model achieved an accuracy of 72.7% in the test set (Figure 3B). The heatmap displays the variation of the selected model features across different samples (Figure 3C), while the boxplot illustrates the differences in data distribution of these peptides between NS and NNS groups (Figure 3D, Supplementary Table S2).

### Potential biomarkers distinguishing NS from other brain diseases

**Figure 4:**
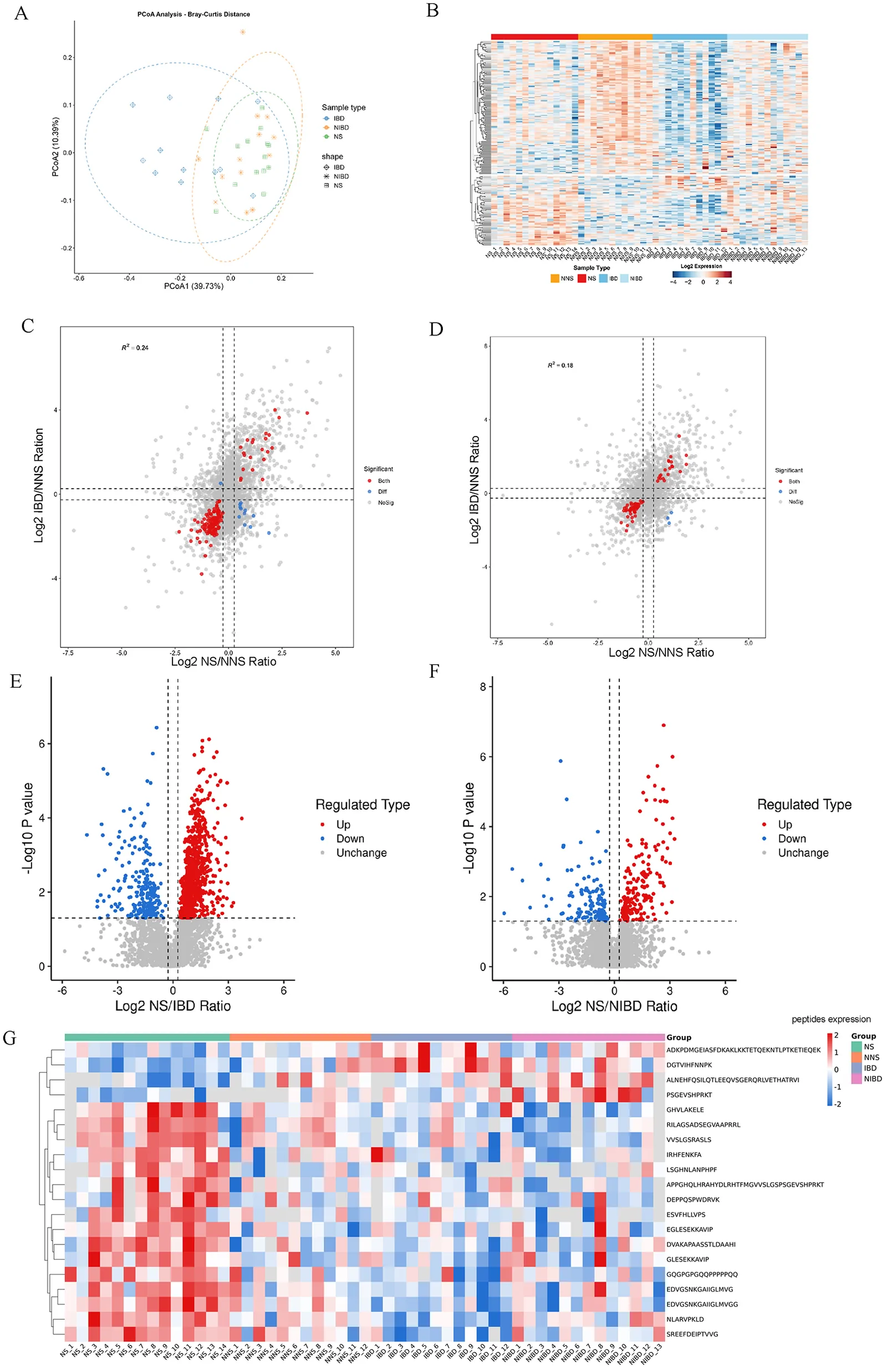
Refinement of NS-specific peptide signatures across neurological conditions. **(A)** PCoA analysis of the peptidomic profile of NS (*n* = 14) IBD (*n* = 12) and NIBD samples (*n* = 13). **(B)** Heatmap showing the ability of differentially expressed peptides between NNS and NS (255 total peptides, z score-normalized log2-transformed value) in CSF among IBD and NIBD samples. The red and blue hollow squares represent peptides with high and low identified abundance in the four groups, respectively. **(C)** Scatter plot comparing the log-fold change of NS vs. NNS (x-axis) against the log-fold change of IBD vs. NNS (y-axis). Red dots represent peptides with a consistent direction of change and a *p*-value < 0.05 in both pairwise comparisons. Blue dots represent peptides with opposite direction of change and a *p*-value < 0.05 in both pairwise comparisons. **(D)** Scatter plot comparing the log-fold change of NS vs. NNS (x-axis) against the log-fold change of NIBD vs. NS (y-axis). Red dots represent peptides with a consistent direction of change and a *p*-value < 0.05 in both pairwise comparisons. Blue dots represent peptides with opposite direction of change and a *p*-value < 0.05 in both pairwise comparisons. Volcano plots depict –log10 (*p*-value) plotted against log_2_ fold changes in peptides abundance for CSF when comparing **(E)** NS vs. IBD and **(F)** NS vs. NIBD. The limma package was used for pairwise comparisons to identify peptides showing significant expression differences. Those beyond the predefined fold-change threshold (FC cutoff: > 1.2 or < 1/1.2, *P* < 0.05) are shown in red (upregulated) or blue (downregulated). **(G)** Heatmap showing differentially expressed peptides in the CSF between NS (*n* = 14), NNS (*n* = 12), IBD (*n* = 12) and NIBD (*n* = 13) samples. Rows of peptides with more than one-third missing values and rows with zero standard deviation were removed when generating the heatmap.

To further evaluate whether the 255 differentially expressed peptides identified between the NS and NNS groups could discriminate NS from other brain disorders, CSF samples were additionally collected from 12 IBD patients and 13 NIBD patients for peptidomic analysis. The PCoA demonstrated an overall separation among the three groups (IBD, NIBD, and NS). However, partial overlap among the clusters suggested that the peptide profiles of NS patients shared certain similarities with those of IBD and NIBD patients (Figure 4A). HCA further revealed that the peptide expression patterns of NS patients were more similar to those of NIBD patients (Figure 4B). Correlation analysis of peptide co-expression patterns showed that the similarity between NS and NIBD patients was relatively low (*R*^2^ = 0.18; Figure 4C), whereas a slightly higher correlation was observed between NS and IBD patients (*R*^2^ = 0.24; Figure 4D). Comprehensive analysis of the 255 differentially expressed peptides between the NS and NNS groups identified 54 peptides capable of distinguishing NS from IBD (Figure 4E) and 50 peptides capable of distinguishing NS from NIBD (Figure 4F). Importantly, 22 peptides were consistently identified as potential biomarkers capable of discriminating NS from NNS, IBD, and NIBD simultaneously (Figure 4G).

### Biomarker verification through PRM-MS

**Figure 5:**
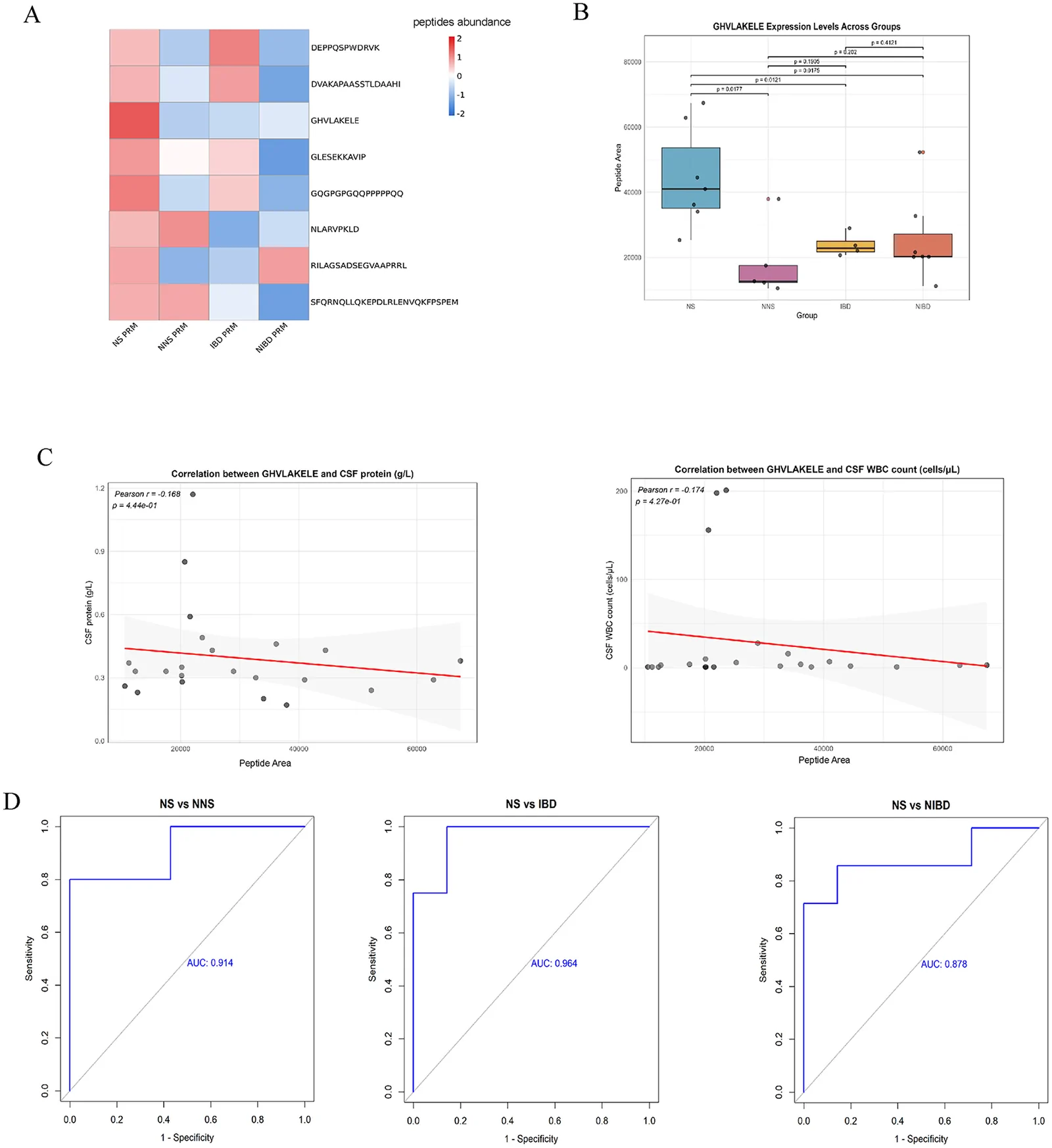
PRM-MS verification of GHVLAKELE (IGF2-derived peptide) as a diagnostic biomarker for neurosyphilis. **(A)**. Heatmap showing peptides abundance in the CSF between NS (*n* = 7), NNS (*n* = 5), IBD (*n* = 4) and NIBD (*n* = 7) groups. **(B)**. Boxplot showing the abundance of GHVLAKELE in CSF samples from NS (*n* = 7), NNS (*n* = 5), IBD (*n* = 4), and NIBD (*n* = 7) groups. Peptide abundance was quantified by PRM-MS. Each dot represents an individual sample. Horizontal lines indicate medians; boxes represent interquartile ranges; whiskers extend to the minimum and maximum values. **(C)**. Scatter plots and linear regression analyses of GHVLAKELE Peptide Area vs. CSF protein and WBC count. **(D)**. ROC curves evaluating the diagnostic performance of GHVLAKELE. The diagonal dashed line represents random classification (AUC = 0.5).

To further identify reliable neurosyphilis biomarkers, we validated 27 candidate peptides (selected based on machine learning scores and differential expression) using PRM-MS (Supplementary Table S3). Next, we collected 23 CSF samples, including 7 NS, 5 NNS, 4 IBD and 7 NIBD. Heatmap shown candidate peptides abundance in the CSF between NNS, NS, IBD, and NIBD groups by PRM (Figure 5A).

GHVLAKELE was identified as a potential biomarker by PRM. The precursor protein is IGF2, according to the canonical human IGF2 precursor sequence (UniProtKB P01344), GHVLAKELE corresponds to residues 139–147 of the 180-amino-acid precursor. (Supplementary Figure S1). The level of GHVLAKELE in the CSF of NS is significantly higher than that in NNS, IBD and NIBD (Figure 5B). The level of GHVLAKELE shows no consistent correlation with CSF protein concentration or white blood cell count (Figure 5C). ROC curve analysis was performed to evaluate the discriminative ability of GHVLAKELE for three pairwise comparisons. The AUC was 0.914 [95% Confidence Interval (CI): 0.728–1.000, *P* < 0.05] for distinguishing NS from NNS, 0.964 (95% CI: 0.865–1.000, *P* < 0.05) for NS from IBD, and 0.878 (95% CI: 0.667–1.000, *P* < 0.05) for NS from NIBD (Figure 5D). These results indicate encouraging diagnostic potential accuracy for NS vs. IBD, good accuracy for NS vs. NNS, and fair-to-good performance for NS vs. NIBD. Collectively, the tested marker demonstrated strong discriminative capacity across all three comparisons (all AUCs > 0.85), suggesting its potential utility as an adjunctive tool for differentiating these clinical conditions.

## Discussion

In this study, we provide a systematic characterization of the CSF peptidome in neurosyphilis and identify peptide signatures associated with disease status. By integrating comparative analysis, machine learning, and targeted validation, we demonstrate that peptidomic profiling can distinguish NS from both syphilis and other neurological conditions.

Recent advances in neurosyphilis biomarker research have predominantly focused on cytokine profiles, protein-level changes, and novel sample types. Zhao et al. measured forty-eight cytokines/chemokines in the CSF samples and found that IL-2Rα and IP-10 are promising predictive biomarkers for neurosyphilis progression, and that their combined use achieves high diagnostic accuracy in distinguishing symptomatic from asymptomatic disease (Zhao et al., 2026). Tang et al. (2026) performed CSF proteomic analysis and identified dysregulation of lysosomal proteins (PSAP, CTSL, NPC2, DNASE2) and axonal guidance proteins (EPHA4) in neurosyphilis patients, replicating previously reported biomarkers SEMA7A, SERPINA3, and ITIH4. More recently, Zhang et al. applied Mendelian randomization to identify METAP2 as a key molecule through which *T. pallidum* inhibits CD4^+^ T-cell proliferation (Liu et al., 2025). While these studies have significantly advanced our understanding of neurosyphilis pathogenesis and provided valuable diagnostic candidates, peptidomics offers distinct and complementary advantages: (i) it provides an unbiased, global survey of the low-molecular-weight proteome without predefined targets (Palanski et al., 2022); (ii) endogenous peptides often exhibit greater stability in biofluids than intact proteins, making them robust analytes for clinical detection (Neves et al., 2021); (iii) the peptide repertoire can reflect disease-specific cleavage patterns, yielding signatures with high specificity for a given condition (Huan et al., 2022); and (iv) peptidomics can identify novel fragments not captured by conventional proteomics, thereby expanding the biomarker pool. By applying this approach to neurosyphilis for the first time, our study complements recent proteomic findings and reveals a panel of CSF peptides, including the IGF2-derived GHVLAKELE, that distinguishes NS from its clinical mimics with high specificity.

A key finding of our study is the stark contrast in the biological processes associated with the differentially expressed peptides in NS. The upregulation of peptides derived from proteins involved in axon regeneration and the positive regulation of MAPK cascades suggests an active, albeit potentially ineffective, host repair response to CNS injury. The MAPK pathway is crucial for neuronal survival, plasticity, and inflammation, and its activation here may reflect a response to the spirochetal insult (Johnson and Lapadat, 2002; Kato et al., 2013; Xue et al., 2024). Conversely, the strong signature of Alzheimer's disease (AD)-related pathology among the downregulated peptide precursors is particularly striking. The association with positive regulation of amyloid-beta formation (Viles, 2023), neurofibrillary tangle assembly (Moloney et al., 2021; Islam et al., 2025), and tau-protein kinase activity (Stefanoska et al., 2022; Courade et al., 2025) implies that *T. pallidum* infection may initiate or accelerate neurodegenerative cascades reminiscent of AD. This finding aligns with the historical concept of “general paresis” and provides a potential molecular link between chronic infection and the neurodegenerative symptoms observed in late-stage NS. It suggests that the cognitive decline in NS may not be solely due to direct spirochetal damage but also involve alterations in peptide generation, processing, or clearance that may accompany CNS infection and neuroinflammation. From a diagnostic perspective, our multi-stage comparison strategy proved effective. The 255 peptides that differentiated NS from NNS were essential for identifying changes specific to CNS invasion, as opposed to syphilis. However, the true diagnostic challenge lies in distinguishing NS from other brain disorders, which often present with similar clinical and laboratory findings. By comparing the NS-specific peptide panel against samples from IBD and NIBD patients, we identified a refined set of 22 peptides that were differentially abundant between NS and the other two groups. The verification of one such peptide, GHVLAKELE from IGF2, by PRM-MS in an independent cohort is a crucial step toward clinical translation. IGF2 is a major brain growth factor involved in neurodevelopment, synaptic plasticity, and cognitive function (Bracko et al., 2012). Dysregulation of the IGF2 pathway has been implicated in various brain diseases, including Alzheimer's disease, Parkinson's disease, Huntington's disease, and amyotrophic lateral sclerosis (Chen et al., 2011; Sepúlveda et al., 2022; Alberini, 2023). However, it is critical to emphasize that while IGF2 plays important roles in these neurodegenerative disorders, our findings demonstrate that GHVLAKELE, an IGF2-derived peptide has high diagnostic specificity for neurosyphilis, effectively distinguishing it from other infectious and non-infectious neurological conditions. The mechanism underlying the increased abundance of GHVLAKELE in CSF remains unclear. Because GHVLAKELE is located within the C-terminal propeptide/E-domain region of the IGF2 precursor, its increased abundance may reflect alterations in precursor processing, peptide generation or clearance. However, the present study did not quantify intact IGF2 or directly assess protease activity, and therefore cannot determine whether the observed peptide elevation results from enhanced proteolytic generation. In addition, changes in blood-brain barrier permeability may potentially influence CSF peptide abundance but were not directly assessed in this study. Further mechanistic studies are required to determine whether this peptide is a direct byproduct of pathogen-host interaction or a marker of a distinct pathological subtype of NS. The specificity of this peptide for neurosyphilis, as demonstrated by its ability to distinguish NS from both IBD and NIBD (including patients with other neurodegenerative processes), suggests that its generation is linked to the unique pathobiological fingerprint of *T. pallidum* infection. This finding transforms GHVLAKELE from a mere biomarker of neuronal distress.

While our random forest model identified several high-performing peptide combinations, the current study prioritized the validation of a single candidate, GHVLAKELE, which emerged from a conservative multi-stage differential filtering strategy. This approach was chosen to minimize the risk of overfitting and to ensure biological interpretability. The machine learning-derived panels, although promising, require further technical optimization for PRM-MS validation (e.g., synthesis of isotopically labeled standards for low-abundance peptides). Future work will systematically evaluate these panels to potentially construct a multi-peptide diagnostic model with superior accuracy.

While our findings provide proof-of-concept evidence for the utility of CSF peptidomics in neurosyphilis diagnosis, several limitations must be acknowledged candidly. First, the validation cohort (*n* = 23) was relatively small and recruited from a single tertiary referral center, which may limit the generalizability of our results. We acknowledge that the stringent inclusion and exclusion criteria applied in this study—including the exclusion of HIV co-infection, prior antibiotic treatment, immunocompromising conditions, and other confounding CNS disorders—were necessary to minimize biological heterogeneity and ensure the specificity of the peptidomic signatures identified. However, we recognize that these rigorous selection criteria inevitably contributed to the modest sample size of the validation cohort. Although the diagnostic performance of GHVLAKELE remained robust across all comparisons, we recognize that larger, multi-center studies are essential to confirm its clinical utility across diverse populations and to establish population-specific reference intervals. Second, the present study was designed to identify host-derived CSF peptide biomarkers associated with neurosyphilis, rather than to detect pathogen-derived peptides. Accordingly, our database searches were restricted to the Homo sapiens reference proteome. We acknowledge that inclusion of the *Treponema pallidum* protein sequence database in future analyses could enable the identification of pathogen-derived peptides, which may provide complementary biological information and potentially serve as direct markers of spirochetal presence in the CNS. Third, although our random forest model identified multiple candidate peptides with high discriminative power, the present study focused on validating a single marker (GHVLAKELE) to minimize the risk of overfitting and to ensure biological interpretability. We acknowledge that a multi-peptide panel could potentially offer superior diagnostic accuracy, particularly in borderline cases, and our ongoing work is directed toward systematically evaluating combinatorial models using PRM-MS with isotopically labeled internal standards, followed by validation in independent cohorts. Fourth, the current assay relies on mass spectrometry, which is not widely available in routine clinical laboratories. We view this as a translational bottleneck and are actively developing a rapid, high-throughput immunoassay [e.g., Enzyme-Linked Immunosorbent Assay (ELISA) or chemiluminescence-based test] targeting GHVLAKELE, which would facilitate broader implementation in primary and secondary care settings. Preliminary efforts have identified suitable antibody pairs, and assay development is underway. Fifth, the biological clearance rate and half-life of GHVLAKELE in CSF remain unexplored. Longitudinal sampling before and after antibiotic treatment will be required to determine whether this peptide decays with clinical resolution and whether it could serve as a pharmacodynamic marker for treatment response. Another limitation is that the present study measured the endogenous peptide GHVLAKELE but did not simultaneously quantify intact IGF2 or other IGF2 precursor-derived products. Therefore, the increased abundance of GHVLAKELE cannot be unequivocally attributed to enhanced proteolytic processing of IGF2. In addition, blood-brain barrier integrity was not directly assessed because the CSF/serum albumin quotient was not available in the present cohort. Consequently, altered barrier permeability cannot be excluded as a potential contributor to the observed CSF peptide abundance. Future studies should integrate targeted quantification of intact IGF2 and IGF2-derived processing products, assessment of relevant protease activity, and measurement of the CSF/serum albumin quotient to clarify the biological origin and mechanisms underlying GHVLAKELE elevation in neurosyphilis. These investigations are planned as part of our prospective follow-up cohort. Collectively, while these limitations temper the immediate clinical translation of our findings, they do not detract from the robust discriminative capacity of GHVLAKELE observed in this study, and they outline a clear roadmap for subsequent validation and implementation efforts.

In conclusion, our work establishes CSF peptidomics as a powerful tool for investigating neurosyphilis. The distinct peptide signatures we identified not only differentiate NS from its clinical mimics but also reveal a previously underappreciated link between treponemal infection and molecular pathways associated with neurodegeneration and synaptic dysfunction. Most importantly, we have identified an IGF2-derived peptide, GHVLAKELE, that serves as a specific diagnostic marker for neurosyphilis—a finding that distinguishes our work from the extensive literature on IGF2 in Alzheimer's disease, Parkinson's disease, and other neurodegenerative disorders where such specific peptide fragments have never been reported. IGF2-derived peptide represents a significant step toward a more accurate and timely diagnosis of neurosyphilis. This could lead to improved patient outcomes by enabling earlier intervention and avoiding unnecessary treatment, while also opening new avenues for understanding the pathogenesis of this re-emerging infectious disease.

## Conclusion

In summary, this study provides the first systematic peptidomic characterization of the CSF in neurosyphilis. By integrating high-resolution LC-MS/MS, machine learning, and targeted validation, we demonstrate that CSF peptidomic profiling can effectively distinguish neurosyphilis from syphilis, infectious brain diseases without syphilis, and non-infectious neurological disorders without syphilis. The identification of the IGF2-derived peptide GHVLAKELE as a candidate biomarker with high diagnostic accuracy (AUC > 0.85 for all comparisons) represents a key translational finding, holds promise for improving diagnostic precision and may guide earlier intervention. Future studies are warranted to validate these findings in larger, multi-center cohorts and to explore the functional roles of these peptides in neurosyphilis pathophysiology. Moreover, the observed enrichment of peptides associated with amyloid-beta formation, tau pathology, and synaptic dysfunction suggests a potential molecular link between *T. pallidum* infection and neurodegenerative cascades, offering new insights into the pathogenesis of late-stage neurosyphilis.

## Data Availability

The datasets presented in this study can be found in online repositories. The names of the repository/repositories and accession number(s) can be found in the article/Supplementary material.

## Supplementary material

Supplementary Figure S1 Location of the endogenous peptide GHVLAKELE within the human IGF2 precursor. GHVLAKELE corresponds to residues 139–147 of the canonical 180-amino-acid human IGF2 precursor (UniProtKB P01344).

Supplementary Table S1-1 Overview of the characteristics of CSF samples from NS, NNS, IBD, and NIBD patients included in the DIA-MS-based peptidomics experiment in this study. NNS, syphilis/non-neurosyphilis; NS, neurosyphilis; NIBD:non-infectious brain disorders without syphilis; IBD, infectious brain diseases without syphilis.

Supplementary Table S1-2 Overview of the characteristics of CSF samples from NS, NNS, IBD, and NIBD patients involved in PRM-MS experiment in the study. NNS, syphilis/non-neurosyphilis; NS, neurosyphilis; NIBD, non-infectious brain disorders without syphilis; IBD, infectious brain diseases without syphilis.

Supplementary Table S2-1 All peptides identified in CSF samples from NNS, NS, IBD, and NIBD patients by DIA-MS-based peptidomics experiment. NNS, syphilis/non-neurosyphilis; NS, neurosyphilis; NIBD, non-infectious brain disorders without syphilis; IBD, infectious brain diseases without syphilis.

Supplementary Table S2-2 Differentially expressed peptides (totally 225 peptides) identified by DIA-MS-based peptidomics experiment in CSF between NNS (n = 12) and NS (n = 14) samples. The significant differentially expressed peptides were defined as those with a fold change (FC) > 1.2 or < 1/1.2, and a P-value < 0.05.

Supplementary Table S2-3 Differentially expressed peptides (totally 1027 peptides) identified by DIA-MS-based peptidomics experiment in CSF between IBD (n = 12) and NS (n = 14) samples. The significant differentially expressed peptides were defined as those with a fold change (FC) > 1.2 or < 1/1.2, and a P-value < 0.05.

Supplementary Table S2-4 Differentially expressed peptides (totally 306 peptides) identified by DIA-MS-based peptidomics experiment in CSF between NIBD (n = 13) and NS (n = 14) samples. The significant differentially expressed peptides were defined as those with a fold change (FC) > 1.2 or < 1/1.2, and a P-value < 0.05.

Supplementary Table S2-5 Heatmap showing differentially expressed peptides in the CSF between NNS (n = 14), NS (n = 12), IBD (n = 12) and NIBD (n = 13) samples.
